# Human embryonic stem cells cultured on hydrogels grafted with extracellular matrix protein‐derived peptides with polyethylene glycol joint nanosegments

**DOI:** 10.1049/nbt2.12091

**Published:** 2022-10-06

**Authors:** Abdullah A. Alarfaj, Abdurahman H. Hirad, Murugan A. Munusamy, S. Suresh Kumar, Akon Higuchi

**Affiliations:** ^1^ Department of Botany and Microbiology King Saud University Riyadh Saudi Arabia; ^2^ Department of Biotechnology Bharath Institute of Higher Education and Research Chennai‐73 India; ^3^ Department of Chemical and Materials Engineering National Central University Taoyuan Taiwan; ^4^ Department of Reproduction National Center for Child Health and Development Tokyo Japan; ^5^ School of Ophthalmology and Optometry The Eye Hospital of Wenzhou Medical University Wenzhou Medical University Wenzhou Zhejiang China; ^6^ R&D Center for Membrane Technology Chung Yuan Christian University Taoyuan Taiwan

## Abstract

Human pluripotent stem cells (hPSCs) can be proliferated on completely synthetic materials under xeno‐free cultivation conditions using biomaterials grafted with extracellular matrix protein (ECM)‐derived peptides. However, cell culture biomaterials grafted with ECM‐derived peptides must be prepared using a high concentration of peptide reaction solution (e.g. 1000 μg/ml), whereas the ECM concentration of the ECM‐coated surface for hPSC culture is typically 5 μg/ml. We designed a polyethylene glycol (PEG) joint nanosegment (linker) to be used between base cell culture biomaterials and bioactive ECM‐derived peptides to enhance the probability of contact between ECM‐derived peptides and cell binding receptors of hPSCs. Vitronectin‐derived peptides with glycine joint nanosegments (GCGG) were conjugated onto poly (vinyl alcohol‐co‐itaconic acid) hydrogels via PEG joint nanosegments, and human embryonic stem cells (hESCs) were cultivated on these hydrogels. hESCs could successfully be cultivated on hydrogels while maintaining their pluripotency and differentiation potential to differentiate into cells that are induced from three germ layers in vitro and in vivo, where only a 50 μg/ml ECM‐derived peptide concentration was used when the PEG joint nanosegments were introduced into peptides that were grafted onto hydrogel surfaces. The joint nanosegments between bioactive peptides and base cell culture biomaterials were found to contribute to efficient hESC attachment and proliferation.

## 1 INTRODUCTION

Human embryonic stem cells (hESCs) and induced pluripotent stem cells (hiPSCs) are promising cell sources of cell therapy for patients with defects in their organs and tissues. Human ESCs and hiPSCs (human pluripotent stem cells, hPSCs) have higher differentiation abilities than that of adult stem cells, such as bone marrow‐derived stem cells or adipose‐derived stem cells. However, there is a critical issue that hESCs and hiPSCs cannot proliferate on conventional tissue cultivation polystyrene (TCP) dishes, but the cells should be cultured on specific biomaterials, such as extracellular matrix protein (ECM)‐coated dishes or ECM‐derived peptide‐immobilised plates or hydrogels. For ECM‐coated dishes, recombinant vitronectin [1, 2, 3, 4, 5] and recombinant laminin (laminin‐521 or laminin‐511) [6, 7, 8, 9, 10] are selected as coating materials. ECM‐derived peptide‐immobilised dishes are also attractive cell culture materials for hPSCs. This is because the cell culture dishes can be made using completely synthetic materials. On the other hand, ECM should be manufactured from the extraction of animal cells or bacteria for the preparation of ECM‐coated dishes, indicating that ECM‐derived peptide‐immobilised dishes are safer for clinical application than ECM‐coated dishes because of the lower levels of contamination by animal‐derived materials. Furthermore, ECM‐derived peptides can be easily grafted onto hydrogels, of which elasticity can be controlled. Therefore, ECM‐derived peptide‐immobilised dishes having controlled elasticity can be easily provided as cell culture biomaterials for hPSC culture and differentiation.

Several researchers have developed ECM‐derived peptide‐immobilised dishes for the proliferation of hPSCs [11, 12, 13, 14, 15, 16, 17, 18, 19, 20, 21, 22, 23, 24, 25, 26]. Melkoumian et al. reported for the first time that ECM‐derived peptide‐grafted surfaces can support the expansion of hPSCs for over 12 passages [21]. In particular, hPSCs could be extensively cultivated on acrylate surfaces grafted with vitronectin‐derived peptide (VN, KGGPQVTRGDVFTMP) and bone sialoprotein‐derived peptide (BSP, KGGNGEPRGDTYRAY), where the peptide solution should be used at a relatively high concentration of approximately 1000 μg/ml. Melkoumian et al. did not study the effects of glycine oligomer joint nanosegments (linker) or polyethylene glycol (PEG) joint nanosegments extensively.

Klim et al. developed a self‐assembled monolayer (SAM) surface using peptide‐immobilised alkanethiol molecules [22]. They identified 18 different peptides that can be attached to hESCs. Human ESCs could adhere and proliferate on SAM surfaces prepared with vitronectin‐derived peptide (GKKQRFRHRNRKG), BSP (FHRRIKA) and heparin‐binding peptides derived from fibronectin (GWQPPARARI). In this research, Klim et al. did not use a glycine oligomer joint nanosegment or PEG joint nanosegment on peptides.

High‐throughput screening of several peptide designs for the attachment of hPSC‐derived cardiomyocytes was reported by Jia et al. [25] because hPSC‐derived cardiomyocytes have poor adhesion characteristics on cell culture biomaterials. They prepared hydrogel microarrays immobilised with peptides designed from laminin. The hydrogel surface immobilised with peptide taken from laminin‐*β*4 (PMQKMRGDVFSP) with a joint nanosegment of four glycines (GGGG) expressed the highest adhesion of hPSC‐derived cardiomyocytes [25]. However, they used glycine peptide joint nanosegments and did not use polyethylene glycol (PEG) joint nanosegments in the peptide design.

In our previous study [17, 19], we developed poly (vinyl alcohol‐co‐itaconic acid) (PVI) hydrogels with optimal elasticity where several designs (single chain, single chain with joint nanosegments (linkers), dual chain with joint nanosegments and branched‐type chain) of vitronectin‐derived peptide (KGGPQVTRGDVFTMP) with a relatively high concentration (1000 μg/ml) of peptide solution were used in this research. We found that hydrogels grafted with single or dual chains with joint nanosegments supported hPSCs for a long time (over 10 passages) where the joint nanosegment used in this study comprised four glycines, and the PEG joint nanosegment was not used in this study.

In another study in our group [11], we designed and prepared PVI hydrogels grafted with several laminin‐derived peptides with glycine joint nanosegments using a 1000 μg/ml peptide solution concentration. PVI hydrogels grafted with LB2CK peptides (GCGGKGGPMQKMRGDVFSP), which were derived from laminin‐*β*4, showed high hPSC expansion for a long time (over 10 passages) and efficient differentiation into the cells induced from three germ layers as well as cardiomyocytes where the joint nanosegment used in this study comprised GCGG, and the PEG joint nanosegment was not used in this study.

It should be noted that we need to use a high peptide solution concentration for immobilisation on the surface for the preparation of peptide‐grafted dishes for hPSC culture, typically over 1000 μg/ml [11, 17, 19, 21, 25], whereas the ECM‐coating solution concentration is typically 5–20 μg/ml. For example, the recommended coating solution concentrations of recombinant vitronectin and laminin‐521 are both 5 μg/ml for hPSC culture dishes. Typically, an approximately 200 times higher concentration of peptide solution is necessary to graft peptides onto the surface compared to the coating of the protein on the cell culture surface in general [11, 17, 19, 21, 25]. This should be because the matching of peptides with receptors of hPSCs is not sufficient for the peptide‐grafted surface. Therefore, we designed a long PEG joint nanosegment to be used between the hydrogel surface and bioactive peptides to enhance the matching of bioactive peptides with cell‐binding receptors of hESCs in this study. We first prepared PVI hydrogels grafted with PEG joint nanosegments (linker) and subsequently bound vitronectin‐derived peptides (VN4C, GCGGKGGPQVTRGDVFTMP) using a relatively low concentration of peptide solution (50–500 μg/ml), where bioactive peptides include glycine joint nanosegments comprising GCGG and serine, which promote dual chain morphology. We will evaluate the hESC expansion on PVI hydrogels grafted with bioactive peptides via PEG joint nanosegments where the bioactive peptide concentration for the grafting was reduced to 50–500 μg/ml in this investigation and discuss the effect of the long PEG joint nanosegments (linkers) on the hPSC expansion on hydrogels grafted with bioactive peptides.

## 2 METHODS

### 2.1 Ethical statement

Our experiments were approved by the ethics committees of the National Central University (NCU‐109‐010) and Taiwan Land seed Hospital (LHISIRB No. 18‐009‐A2). We followed every relevant governmental and university guideline and regulation.

### 2.2 Materials

Human ESCs (WA09) were obtained from the WiCell Research Institute (Madison, WI, USA). The chemicals, polymer and biomacromolecules utilised in this study are tabulated in Table S1. The other materials utilised in this project were received from Sigma‐Aldrich (St. Louis, MO, USA).

### 2.3 Preparation processes of hydrogels grafted with vitronectin‐derived peptides via PEG joint nanosegments

**FIGURE 1:**
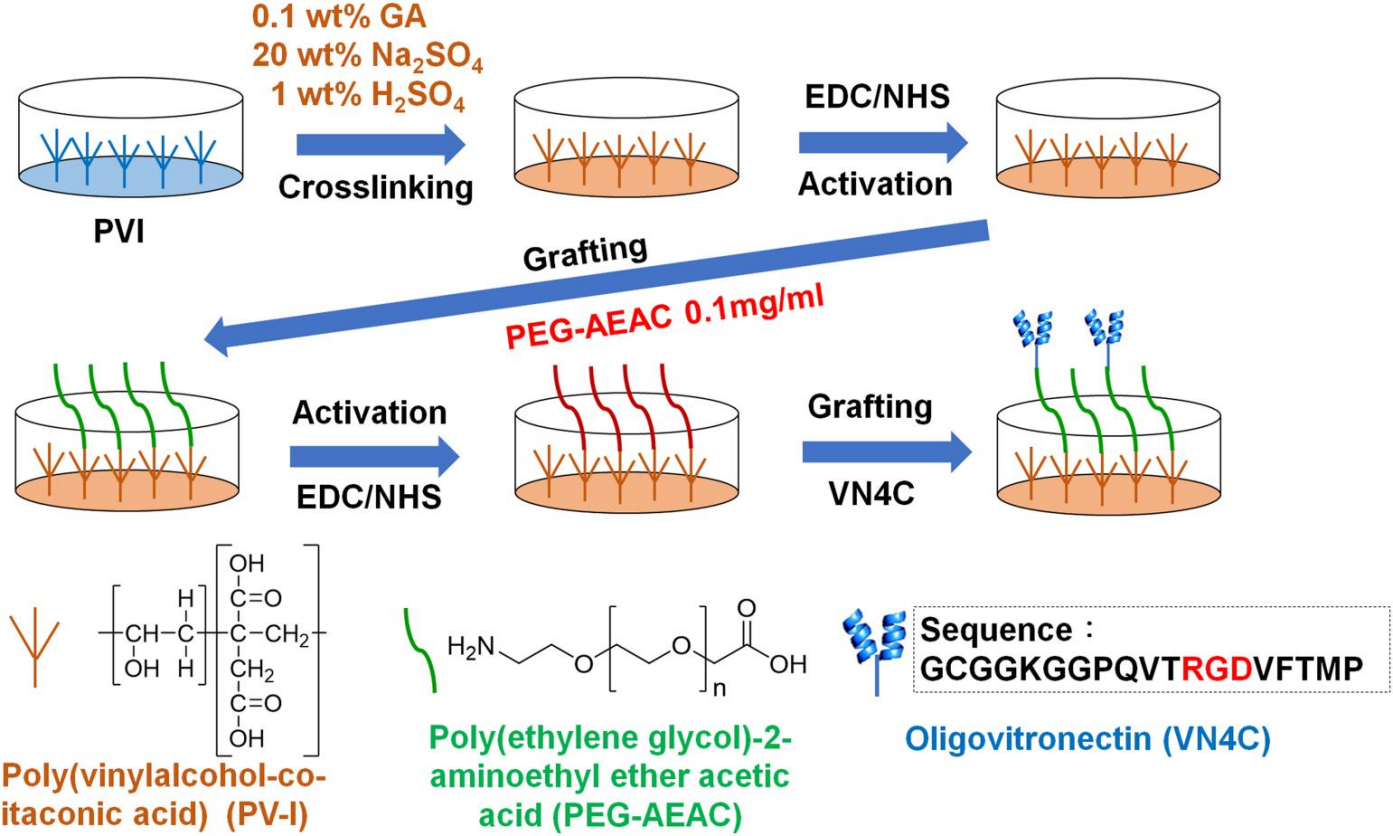
Outline of the preparation method of the poly (vinyl alcohol‐co‐itaconic acid) (PVI) hydrogels grafted with VN4C (GCGGKGGPQVTRGDVFTMP) via polyethylene glycol (PEG) joint nanosegments

Poly (vinyl alcohol‐co‐itaconic acid) (PVI) hydrogels were used as a base cell culture surface where vitronectin‐derived peptides (VN4C, GCGGKGGPQVTRGDVFTMP) were grafted with and without PEG joint nanosegments. The outline of the preparation method of PVI hydrogels grafted with VN4C via PEG joint nanosegments is shown in Figure 1.

PVI with a 98% degree of hydrolysis and 1.3 mol% itaconic acid was dissolved in ultrapure water to create a 0.05 wt% solution according to previous studies [11, 17, 19]. The crosslinking of PVI hydrogels was performed by immersing PVI films into aqueous glutaraldehyde solution (20 wt% Na_2_SO_4_, 1 wt% H_2_SO_4_ and 1 wt% glutaraldehyde) for 24 h to generate an optimal elasticity of hydrogels for hPSC proliferation [11, 17, 19]. For the grafting of VN4C onto PVI hydrogels without PEG joint nanosegments, PVI hydrogels were immersed in aqueous 10 mg/ml EDC (N‐(3‐dimethylaminopropyl)‐N′‐ethylcarbodiimide hydrochloride) and 10 mg/ml NHS (N‐hydroxysuccinimide) solution for 6 h at 4°C, which was followed by immersion in 50, 200 or 500 μg/ml of VN4C aqueous solution to graft the peptide onto PVI hydrogels. These hydrogels were named PVI‐VN4C(50), PVI‐VN4C(200) and PVI‐VN4C(500), respectively, in this study.

For grafting of VN4C onto PVI hydrogels with PEG joint nanosegments, an aqueous solution (0.1 mg/ml) of PEG joint nanosegments (PEG‐2‐aminoethyl ether acetic acid (PEG‐AEAC)) with a number average molecular weight of 10,000 (repeated unit number = approximately 230) was absorbed into PVI hydrogels at 4°C for 24 h after activation using EDC/NHS as described above. Subsequently, PVI hydrogels with PEG joint nanosegments were activated again using 10 mg/ml EDC/10 mg/ml NHS for 6 h at 4°C, which was followed by immersion in 50, 200 or 500 μg/ml of VN4C aqueous solution to graft the peptide onto PVI hydrogels. These hydrogels were named PVI‐PEG‐VN4C(50), PVI‐PEG‐VN4C(200) and PVI‐PEG‐VN4C(500) in this study.

### 2.4 Characterisation of PVI hydrogels grafted with VN4C

The chemical compositions of PVI hydrogels grafted with VN4C and/or PEG joint nanosegments were characterised by XPS (X‐ray photoelectron spectroscopy, K‐Alpha spectrometer, Thermal Scientific, Inc., Amarillo, TX, USA). The binding energy scale was standardised for the peak maximum of the C 1s spectra at 284.6 eV.

### 2.5 Preparation and cultivation of hESCs on PVI hydrogels grafted with VN4C

Human ESCs (WA09) were cultivated on PVI hydrogels grafted with VN4C or on Matrigel‐coated plates (control experiments) in xeno‐free Essential 8 (E8) media as previously reported [11, 27, 28]. The cells were inoculated with PVI hydrogels grafted with VN4C or on Matrigel‐coated dishes at a seeding density of 5 × 10^4^ cells per cm^2^, and the E8 media were changed every day with fresh E8 media during hESC proliferation.

During hESC proliferation, the following equation was utilised to evaluate the expansion fold change, where the number (No.) of hESCs on the plates before and after culture was counted using flow cytometry (BD Accuri™ C6, BD Biosciences, Franklin Lakes, NJ, USA) [11, 27, 28]:

(1) Expansionfoldchange=(No.ofhESCsontheplatesaftercultivation)/(No.ofhESCsontheplatesbeforecultivation)

The differentiation rate was analysed from the percentage of the cells that did not express SSEA‐4 and was evaluated using flow cytometry after cultivation on the plates [11, 27, 28]:

(2) Differentiationrate(%)=PercentageofthecellsthatdonotexpressSSEA‐4

### 2.6 Characterisation of hESCs

The immunostaining of hESCs for pluripotent proteins Oct3/4, SSEA‐4, Nanog and Sox2 was performed to study the pluripotency of hESCs following a conventional assay [11, 27, 28, 29, 30]. The expression levels of Oct3/4, SSEA‐4, Nanog and Sox2 as well as the nuclei staining results using Hoechst were evaluated under a fluorescence microscope (Eclipse Ti‐U, Nikon Instruments, Inc., Tokyo, Japan).

### 2.7 Embryoid body formation assay

The embryoid body (EB) generation of hESCs was used to evaluate the differentiation potential of hESCs to differentiate into cells induced from three germ layers in vitro. Human ESCs were inoculated onto ultralow attachment culture plates in Essential 6 media to form EBs. After two  weeks of cultivation in suspension, EBs were transferred to a Matrigel‐coated surface, where the cells were incubated for an additional month. Then, the cells were stained with antibodies against all three markers of embryonic germline layers (AFP (endoderm, *α*‐fetoprotein), SMA (mesoderm, smooth muscle actin) and GFAP (ectoderm, glial fibrillary acidic protein)) by applying a conventional assay, and the cells were observed under a fluorescence microscope [11, 27, 28, 29, 30].

### 2.8 Teratoma formation assay

Teratoma formation of hESCs was used to analyse the differentiation potential of hESCs to differentiate into cells induced from three germ layers in vivo. Human ESC pellets were added into the DMEM/F12 medium with Matrigel. Nonobese diabetic/severe combined immunodeficiency (NOD.CB17‐Prkdcscid/JNarl NOD/SCID) mice were subcutaneously transplanted with a total of 5.0 × 10^6^ hESCs. After 6–8 weeks, teratomas were typically generated, then taken out and fixed in a formaldehyde solution. The paraffin‐embedded teratoma was sliced, and the sliced teratomas were stained with haematoxylin and eosin (H & E) following a conventional method [11, 27, 28, 29, 30].

### 2.9 Statistical analysis

The quantitative data were obtained from four samples in this research. The data are shown as the mean ± standard deviation (SD). A statistical analysis was conducted by performing unpaired Student's *t* tests in Excel (Microsoft Corporation). Probability values (*p*) less than 0.05 were considered statistically significant.

## 3 RESULTS AND DISCUSSION

### 3.1 Design and characterisation of PVI hydrogels grafted with VN4C peptide via PEG joint nanosegment

We prepared PVI hydrogels grafted with VN4C peptides (GCGGKGGPQVTRGDVFTMP) where PEG joint nanosegments were conjugated between carboxylic acid groups of PVI hydrogels and amine groups of VN4C peptides (PVI‐PEG‐VN4C(50), PVI‐PEG‐VN4C(200) and PVI‐PEG‐VN4C(500)). We also prepared PVI hydrogels grafted with VN4C without PEG linkers as control hydrogels (PVI‐VN4C(50), PVI‐VN4C(200) and PVI‐VN4C(500)). PEG‐AEAC with a molecular weight = 10,000 (repeated unit number = approximately 230) was selected for use as a PEG joint nanosegment (linker), and VN4C comprised a vitronectin‐derived sequence of KGGPQVTRGDVFTMP as well as a joint nanosegment of GCGG with serine, which contributed to a dual chain morphology.

**FIGURE 2:**
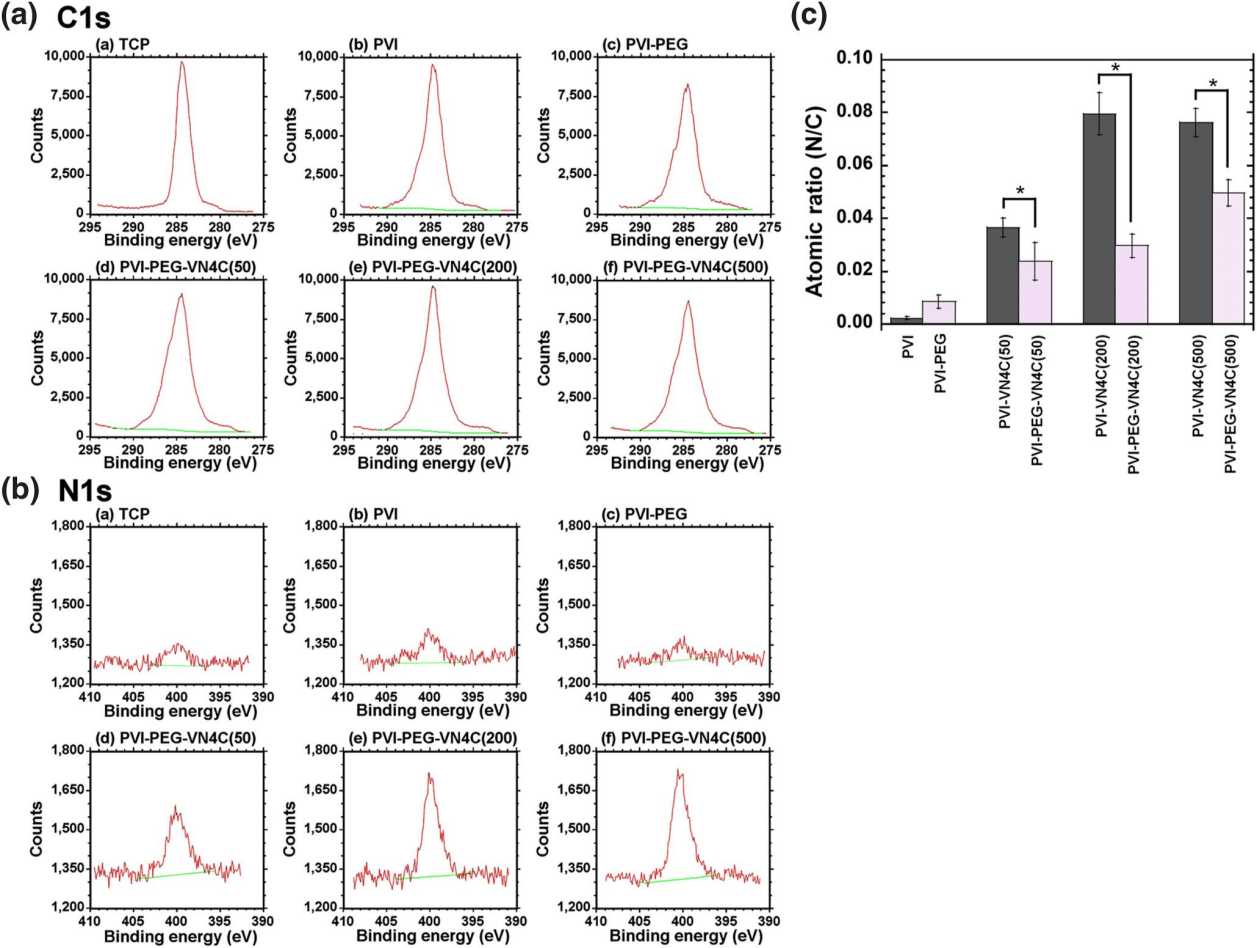
Surface analysis of poly (vinyl alcohol‐co‐itaconic acid) (PVI) hydrogels. (a) High‐resolution X‐ray photoelectron spectroscopy (XPS) spectra of the C 1s peaks on the surfaces of the (a) tissue cultivation polystyrene (TCP) dishes, (b) PVI hydrogels, (c) PVI‐PEG hydrogels, (d) PVI‐PEG‐VN4C(50) hydrogels, (e) PVI‐PEG‐VN4C(200) hydrogels and (f) PVI‐PEG‐VN4C(500) hydrogels. (b) High‐resolution XPS spectra of the N 1s peaks on the surfaces of the (a) TCP dishes, (b) PVI hydrogels, (c) PVI‐PEG hydrogels, (d) PVI‐PEG‐VN4C(50) hydrogels, (e) PVI‐PEG‐VN4C(200) hydrogels and (f) PVI‐PEG‐VN4C(500) hydrogels. (c) The nitrogen to carbon (N/C) atomic ratios on the surfaces of the PVI, PVI‐PEG, PVI‐VN4C and PVI‐PEG‐VN4C hydrogels. *p < 0.05

Before hESC cultivation on PVI hydrogels grafted with VN4C with and without PEG joint nanosegments (linkers), we characterised PVI hydrogels using XPS. This was because we considered that the N/C atomic ratio on hydrogels was proportionally related to the surface density of VN4C on the PVI‐VN4C and PVI‐PEG‐VN4C hydrogels. Figures 2a and 2b show the high‐resolution XPS spectra of the C 1s and N 1s peaks, respectively, of the (a) TCP plates (negative control), (b) nonmodified PVI hydrogels, (c) PVI‐PEG hydrogels, (d) PVI‐PEG‐VN4C(50) hydrogels, (e) PVI‐PEG‐VN4C(200) hydrogels and (f) PVI‐PEG‐VN4C(500) hydrogels. Figure S1A and S1B show the high‐resolution XPS spectra of the C 1s and N 1s peaks, respectively, of (a) PVI‐VN4C(50) hydrogels, (b) PVI‐VN4C(200) hydrogels and (c) PVI‐VN4C(500) hydrogels. Distinct peaks at approximately 285 eV were found in C 1s spectra for all material surfaces investigated in this study. In particular, hydrogels grafted with peptides expressed wider peaks of C 1s spectra than that of the TCP dishes, which is ascribed to the peptide bonding carbons derived from VN4C peptides.

N 1s peaks from peptide bonding were distinctly observed at approximately 400 eV for the PVI‐PEG‐VN4C hydrogels, whereas almost no N 1s peaks were observed for TCP dishes, non‐modified PVI hydrogels and PVI‐PEG hydrogels. This is because no nitrogen atoms exist on TCP dishes, non‐modified PVI hydrogels and PVI‐PEG hydrogels.

The atomic ratios of nitrogen to carbon (N/C) were evaluated for several hydrogels with and without PEG joint nanosegments (linkers) and are depicted in Figure 2c. The N/C atomic ratios of PVI‐VN4C hydrogels were higher than those of PVI‐PEG‐VN4C hydrogels when these hydrogels were prepared with the same peptide concentrations of VN4C. Furthermore, the N/C atomic ratios of the hydrogel surfaces increased with increasing peptide concentration of VN4C in the preparation of PVI‐VN4C and PVI‐PEG‐VN4C hydrogels.

### 3.2 Human ESCs cultured on PVI‐VN4C and PVI‐PEG‐VN4C hydrogels

**FIGURE 3:**
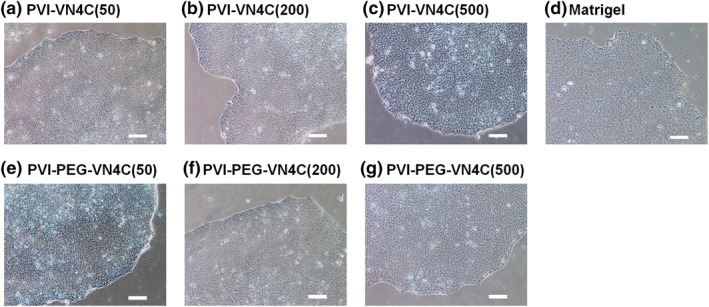
Human ESCs (WA09) cultured on poly (vinyl alcohol‐co‐itaconic acid) (PVI) hydrogels grafted with VN4C peptide. Morphologies of human embryonic stem cells (hESCs) on (a) PVI‐VN4C(50) hydrogels, (b) PVI‐VN4C(200) hydrogels, (c) PVI‐VN4C(500) hydrogels, (d) Matrigel‐coated tissue cultivation polystyrene (TCP) dishes, (e) PVI‐PEG‐VN4C(50) hydrogels, (f) PVI‐PEG‐VN4C(200) hydrogels and (g) PVI‐PEG‐VN4C(500) hydrogels. The scale bar indicates 100 μm

**FIGURE 4:**
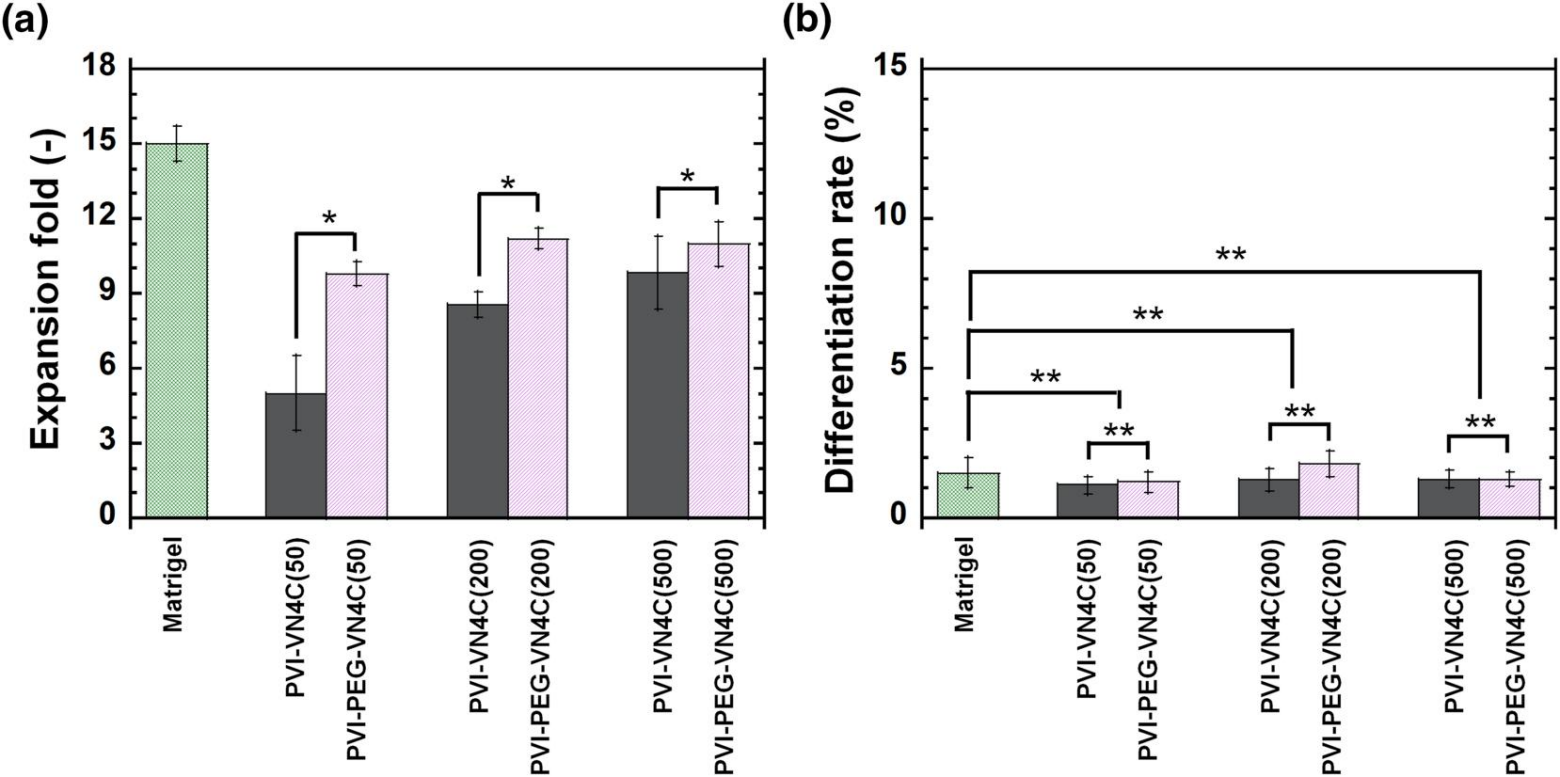
Human ESCs (WA09) cultured on poly (vinyl alcohol‐co‐itaconic acid) (PVI) hydrogels grafted with VN4C peptide. (a) Expansion folds of human embryonic stem cells (hESCs) on Matrigel‐coated tissue cultivation polystyrene (TCP) dishes, PVI‐VN4C hydrogels and PVI‐PEG‐VN4C hydrogels. *p < 0.05. (b) Differentiation rates of hESCs on Matrigel‐coated TCP plates, PVI‐VN4C hydrogels and PVI‐PEG‐VN4C hydrogels. **p > 0.05

Human ESCs were cultured on PVI‐VN4C and PVI‐PEG‐VN4C hydrogels as well as on Matrigel‐coated dishes (control experiments) in xeno‐free E8 medium for one passage (1 week). The morphologies of hESCs on each hydrogel surface were investigated and are shown in Figure 3. hESCs showed good shapes (round shapes) and big colony morphologies under each condition, indicating that hESCs were in healthy condition, although the colony numbers (numbers of hESCs) were different depending on the PVI hydrogel being studied. To investigate which PVI hydrogels grafted with the bioactive peptide VN4C with or without PEG joint nanosegments (linkers) were more effective cell culture biomaterials for the expansion of hESCs, the expansion folds and differentiation rates of hESCs were investigated after one  week of culture, and the results are shown in Figure 4. The expansion fold of hESCs on PVI‐PEG‐VN4C was higher than that of PVI‐VN4C when these hydrogels were prepared with the same peptide concentration of VN4C. There was a tendency for the expansion folds of hESCs on PVI hydrogels to increase with increasing peptide concentration of VN4C in the preparation of PVI‐VN4C and PVI‐PEG‐VN4C hydrogels, and the expansion fold of hESCs on PVI‐PEG‐VN4C(50) was still approximately 10‐fold, where only 50 μg/ml of VN4C was used for the preparation of PVI‐PEG‐VN4C(50) hydrogels. The expansion fold of the hESCs on PVI‐VN4C(50) showed almost half of the expansion fold of hESCs on PVI‐PEG‐VN4C(50). The VN4C peptides on PVI‐PEG‐VN4C(50) and PVI‐VI4C(50) contained glycine joint nanosegments of GCGG. Therefore, the additional linker of the PEG joint nanosegment is beneficial for the attachment and expansion of hESCs on PVI‐PEG‐VN4C(50) hydrogels.

The differentiation rates of hESCs cultured on several cell culture biomaterials (PVI‐VN4C hydrogels, PVI‐PEG‐VN4C hydrogels and Matrigel‐coated dishes) after one  week of culture were investigated and are illustrated in Figure 4b. The differentiation rates of hESCs was extremely low, less than 2%–3%, when cultured on PVI‐VN4C hydrogels, PVI‐PEG‐VN4C hydrogels and Matrigel‐coated dishes, indicating that both PVI‐VN4C hydrogels and PVI‐PEG‐VN4C hydrogels could maintain the same pluripotency of hESCs as the Matrigel‐coated dishes.

**FIGURE 5:**
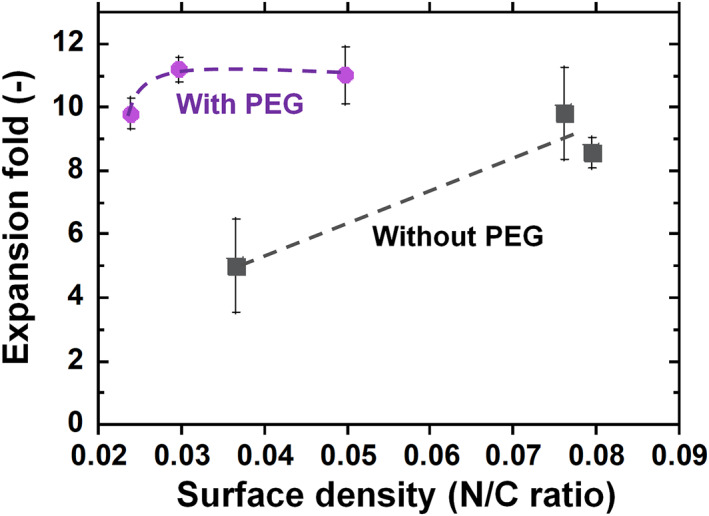
Effect of human embryonic stem cell (hESC) expansion fold on the surface density (N/C atomic ratios) of bioactive peptide VN4C, where hESCs were cultured on poly (vinyl alcohol‐co‐itaconic acid) (PVI)‐VN4C hydrogels and PVI‐PEG‐VN4C hydrogels for one passage

We intended to evaluate the effect of hESC expansion on the surface density of the bioactive peptide VN4C, where hESCs are cultured on PVI‐VN4C hydrogels and PVI‐PEG‐VN4C hydrogels, and the results are shown in Figure 5. It should be noted that the surface density of the bioactive peptide VN4C was extremely difficult to measure because of the nanothickness of the VN4C peptide on surfaces of the prepared PVI‐VN4C and PVI‐PEG‐VN4C hydrogels. Therefore, the N/C atomic ratio was used instead of the surface density of the bioactive peptide VN4C in Figure 5. The expansion fold of hESCs on PVI‐PEG‐VN4C was extensively higher than that on PVI‐VN4C at the same N/C atomic ratio (same surface density of VN4C peptide), indicating that the VN4C on PVI‐PEG‐VN4C hydrogels effectively contributed to hESC adhesion and expansion compared to that on PVI‐VN4C hydrogels.

It should be noted that only a 50 μg/ml bioactive peptide solution concentration was enough for hESCs to adhere and expand on PVI‐PEG‐VN4C hydrogels in this study. The preparation of conventional peptide‐grafted dishes requires a high concentration of peptide. For example, Melkoumian et al. prepared a peptide‐grafted acrylate surface using concentrations greater than 0.5 mM VN (KGGPQVTRGDVFTMP) peptide corresponding to 820 μg/ml and 0.5 mM BSP (KGGNGEPRGDTYRAY) peptide corresponding to 790 μg/ml [21], where the concentration in their study was more than one order of magnitude higher than 50 μg/ml VN4C, which was used for the preparation of PVI‐PEG‐VN4C(50) hydrogels in this study.

Jia et al. prepared PEG hydrogels conjugated with several peptide designs using a 15 mM peptide solution [25]. The best peptide‐conjugated PEG hydrogels for hPSC‐derived cardiomyocyte attachment were prepared using a 15 mM solution of laminin‐*β*4‐derived peptide (PMQKMRGDVFSP) with joint nanosegments comprising four glycines, which corresponded to 24,312 μg/ml, where the concentration in their study was also more than one order of magnitude higher than 50 μg/ml VN4C, which was used for the preparation of PVI‐PEG‐VN4C(50) hydrogels in this work.

We have developed several types of PVI hydrogels grafted with several types of ECM‐derived peptides, [11, 17, 19] which were derived from vitronectin and laminin where the peptide concentration of the reaction solution for the grafting of peptides was selected to be 1000 μg/ml, where the concentration in their study was also more than one order of magnitude higher than 50 μg/ml VN4C, which was used for the preparation of PVI‐PEG‐VN4C(50) hydrogels in this study. Considering these previous results [11, 17, 19, 21, 25], the introduction of PEG joint nanosegments (linkers) between bioactive nanosegments and hydrogels (or dishes) is an extremely powerful design for developing efficient bioactive peptide‐grafted hydrogels, like the ones in this study. However, it should be necessary to confirm the effectiveness of PEG joint nanosegments (linkers) between bioactive nanosegments and hydrogels (or dishes) on hPSC proliferation from longer term hPSC cultivation such as cultivation of hPSCs for over 10 passages in future.

### 3.3 Pluripotency and differentiation potential of hESCs after cultivation on PVI‐PEG‐VN4C hydrogels

**FIGURE 6:**
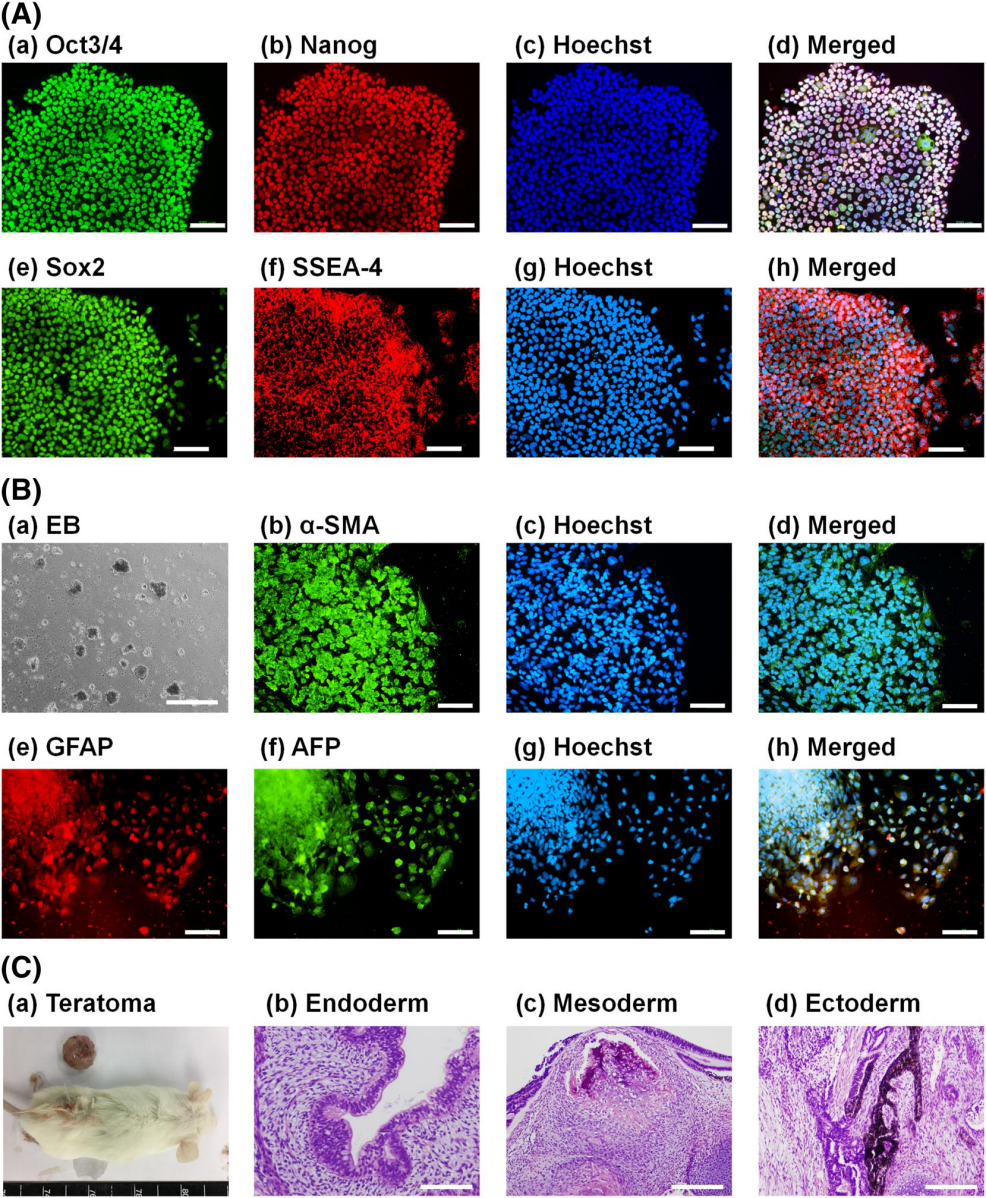
Pluripotency evaluation of human embryonic stem cells (hESCs) after cultivation on PVI‐PEG‐VN4C(50) hydrogels for one passage. (A) Pluripotent protein expressions of Oct3/4 (a, green), Nanog (b, red), Sox2 (e, green), and SSEA‐4 (f, red) in hESCs evaluated by immunohistochemical staining with nuclear staining by Hoechst 33,342 (blue, (c), (g). Images (d) and (h) were made by merging (a)–(c) and (e)–(g), respectively. The scale bar indicates 100 μm. (B) (a) Morphologies of embryoid body (EB) cells differentiated from hESCs. (b)–(h) Expressions of a mesodermal protein marker (b, green, *α*‐SMA), an ectodermal protein marker (e, red, GFAP) and an endodermal protein marker (f, green, AFP) from EB cells analysed by immunohistochemical staining with nuclear staining from Hoechst 33,342 (c, g, blue). Photographs (d) and (h) were made by merging (b)–(c) and (e)–(g), respectively. The scale bar indicates 500 μm for (a) and 100 μm for (b)–(h). (C) (a) A teratoma generated by transplantation of hESCs (H9) into NOD/SCID mice. (b–d) Tissues including glandular ducts composed of cylindrical epithelium (b, endoderm), bone‐like tissue (c, mesoderm) and melanin‐producing cells (d, ectoderm) can be observed. The scale bar indicates 100 μm (b) and 200 μm (c,d)

Human ESCs, which were cultivated on PVI‐PEG‐VN4C hydrogels prepared using the lowest concentration of VN4C solution (PVI‐PEG‐VN4C(50) hydrogels) for one passage, were evaluated to investigate whether hESCs could sustain their pluripotency and hold the potential to induce differentiation into the cells from three germ layers after hESC cultivation for one passage on PVI‐PEG‐VN4C(50) hydrogels in xeno‐free cultivation conditions. Figure 6A illustrates the protein marker expression of pluripotency (Oct3/4, Nanog, Sox2 and SSEA‐4) in hESCs by immunohistochemical analysis after hESC culture on PVI‐PEG‐VN4C(50) hydrogels for one passage in xeno‐free cultivation conditions, where Hoechst 33,342 was used to stain hESCs to describe the nuclei of the cells. All pluripotent marker proteins were expressed in hESCs even after cultivation on PVI‐PEG‐VN4C(50) hydrogels, indicating that hESCs could maintain their pluripotency even after their culture on PVI‐PEG‐VN4C(50) hydrogels in xeno‐free cultivation conditions.

The differentiation ability of hESCs to differentiate into cells induced from three germ layers was also evaluated for hESCs after their culture for one passage on PVI‐PEG‐VN4C(50) hydrogels in xeno‐free cultivation conditions, where an EB formation assay in vitro and teratoma formation in vivo were used in this study. Figure 6b illustrates the morphologies of EBs and the immunohistochemically stained cells that migrated from EBs, which were yielded from hESCs after their culture for one passage on PVI‐PEG‐VN4C(50) hydrogels in xeno‐free culture conditions. The cells that diffused from EBs on Matrigel‐coated dishes showed high expressions of proteins derived from three germ layers: (a) *α*‐SMA (mesoderm cells), (b) GFAP (ectoderm cells) and (c) AFP (endoderm cells).

A teratoma formation assay was performed to evaluate the in vivo abilities of hESCs to differentiate into cells derived from the three germ layers. After one passage cultivation of hESCs on PVI‐PEG‐VN4C(50) hydrogels in xeno‐free culture conditions, hESCs were injected subcutaneously into immunodeficient NOD/SCID (NOD.CB17‐*Prkdc* ^scid^/Jnarl) mice to create teratomas (Figure 6C(a)). Teratomas were then sampled from the NOD/SCID mice, fixed and sectioned. The sliced teratoma samples were stained with haematoxylin and eosin (H&E). Some extensive types of tissue cells were obtained from teratomas, such as the endoderm (glandular ducts composed of cylindrical epithelium, Figure 6C(b)), mesoderm (bone‐like tissue, Figure 6C(c)) and ectoderm (melanin‐producing cells, Figure 6C(d)). Therefore, hESCs could differentiate into cells derived from three germ layers, which indicated that hESCs could sustain their differentiation capability (pluripotency) in vitro and in vivo even after proliferation on PVI‐PEG‐VN4C(50) hydrogels in xeno‐free cultivation conditions.

## 4 CONCLUSION

Human embryonic stem cells could successfully be cultivated on PVI‐PEG‐VN4C(50) hydrogels while maintaining their pluripotency and differentiation ability to differentiate into cells induced from three germ layers in vitro, where only a 50 μg/ml of ECM‐derived peptide solution concentration was used when PEG joint nanosegments were introduced into peptides to be grafted onto the hydrogel surface, whereas conventional cell culture biomaterials grafted with ECM‐derived peptide, which could support hESC proliferation, are generally prepared using peptide reaction solutions with high peptide concentrations, such as approximately 1000 μg/ml [11, 17, 19, 21, 25]. The long PEG joint nanosegment (linker) between the bioactive peptide and the base cell culture biomaterial is important for developing cell culture biomaterials grafted with ECM‐derived peptides, which can contribute to efficient hESC attachment and proliferation.

## CONFLICT OF INTEREST

The authors report no conflict of interest in this work.

## PERMISSION TO REPRODUCE MATERIALS FROM OTHER SOURCES

None.

## Supporting information

Supporting Information S1

## Data Availability

The data that support the findings of this study are available from the corresponding author upon reasonable request.
