# Supplementary material for: Human embryonic stem cells cultured on hydrogels grafted with extracellular matrix protein‐derived peptides with polyethylene glycol joint nanosegments
Source: IET Nanobiotechnol. 2022 Oct 6;16(9):295–304. doi: 10.1049/nbt2.12091 (PMC9667744; doi:10.1049/nbt2.12091)
Supplement: Supplementary file 1 — Supporting Information S1 [file NBT2-16-295-s001.pdf]

Human embryonic stem cells cultured on hydrogels grafted with ECM-derived peptides with PEG joint nanosegments

Abdullah A. Alarfaj [1], Abdurahman H. Hirad [1], Murugan A. Munusamy [1], S. Suresh Kumar [2,*]

and Akon Higuchi [3-6,*]

[1]Department of Chemistry, College of Sciences, King Saud University, Riyadh 11451, Saudi Arabia;

[2]Department of Biotechnology, Bharath Institute of Higher Education and Research, Tambaram East, Chennai-73, 600078, India

[3]Department of Chemical and Materials Engineering, National Central University, Taoyuan 320, Taiwan

[4]Department of Reproduction, National Center for Child Health and Development, Tokyo 157-8535, Japan

[5]School of Ophthalmology and Optometry, The Eye Hospital of Wenzhou Medical University, Wenzhou Medical University, Zhejiang 325027, China

[6]R&D Center for Membrane Technology, Chung Yuan Christian University, Taoyuan 320, Taiwan

* Correspondence author.

S. Suresh Kumar, Department of Biotechnology, Bharath Institute of Higher Education and Research, 173, Agaram Road, Tambaram East, Chennai-73, 600078, India

Email: sureshkudsc@gmail.com

Akon Higuchi, Department of Chemical and Materials Engineering, National Central University, No. 300, Jhongda RD., Jhongli, Taoyuan 320, Taiwan

Email: higuchi@ncu.edu.tw

Supplementary Information

Table S1 Materials used in this study.

Materials	Abbreviation	Catalog No.	Company
		**ECM**	
Matrigel	Matrigel	#356230	Corning (Corning, NY, USA)
Recombinant vitronectin	rVN	A14700	Thermo Fisher Scientific (Waltham, MA, USA)
		**Cell culture dishes**	
6-well polystyrene plate	TCP	#353046	Corning (Corning, NY, USA)
		**Chemicals & Polymer**	
Dispase II	Dispase	D4693-1G	Sigma-Aldrich (St. Louis, MO, USA)
N-hydroxysuccinimide	NHS	13062	Sigma-Aldrich (St. Louis, MO, USA)
N-(3-Dimethylaminopropyl)-N'-ethylcarbodiimide hydrochloride	EDC	3450	Sigma-Aldrich (St. Louis, MO, USA)
PEG-2-aminoethyl ether acetic acid	PEG-AEAC	757705-100MG	Sigma-Aldrich (St. Louis, MO, USA)
Poly(vinyl alcohol-co-itaconic acid)	PVI		PH Japan Co. Ltd. (Hiroshima, Japan)
		**Cell culture medium and component**	
Essential 8 medium	Essential 8	A1517001	Thermo Fisher Scientific (Waltham, MA, USA)
Essential 6 medium	Essential 6	A1516401	Thermo Fisher Scientific Inc. (Waltham, MA, USA)
DMEM/F12 medium	DMEM/F12 medium	11330-057	Thermo Fisher Scientific (Waltham, MA, USA)
RPMI 1640	RPMI 1640	11875093	Thermo Fisher Scientific (Waltham, MA, USA)
Hoechst 33342	Hoechst	PA-3014	Lonza (Basel, Switzerland)
		**Antibodies**	
Anti-Oct3/4 antibody	Anti-Oct3/4 antibody	sc-5279	Santa Cruz Biotechnology (Dallas, TX, USA)
Anti-Sox2 antibody	Anti-Sox2 antibody	AB5603	Merck KGaA (Darmstadt, Germany)
Anti-SSEA-4 antibody	Anti-SSEA-4 antibody	ab16287	Abcam (Cambridge, MA, USA)
Anti-Nanog antibody	Anti-Nanog antibody	MA1-017	Thermo Fisher Scientific (Waltham, MA, USA)
Anti-α-SMA antibody	Anti-α-SMA antibody	PA5-19465	Thermo Fisher Scientific (Waltham, MA, USA)
Anti-GFAP antibody	Anti-GFAP antibody	MA5-15086	Thermo Fisher Scientific (Waltham, MA, USA)
Anti-AFP antibody	Anti-AFP antibody	PA5-21004	Thermo Fisher Scientific (Waltham, MA, USA)
Alexa Fluor 488 goat anti-mouse IgG	Alexa Fluor 488 goat anti-mouse IgG	A-11001	Thermo Fisher Scientific (Waltham, MA, USA)
Alexa Fluor 488 goat anti-rabbit IgG	Alexa Fluor 488 goat anti-rabbit IgG	A-11008	Thermo Fisher Scientific (Waltham, MA, USA)
Alexa Fluor 555 goat anti-mouse IgG	Alexa Fluor 555 goat anti-mouse IgG	A-21424	Thermo Fisher Scientific (Waltham, MA, USA)
Alexa Fluor 555 goat anti-rabbit IgG	Alexa Fluor 555 goat anti-rabbit IgG	ab150078	Abcam (Milton, Cambridge, UK)

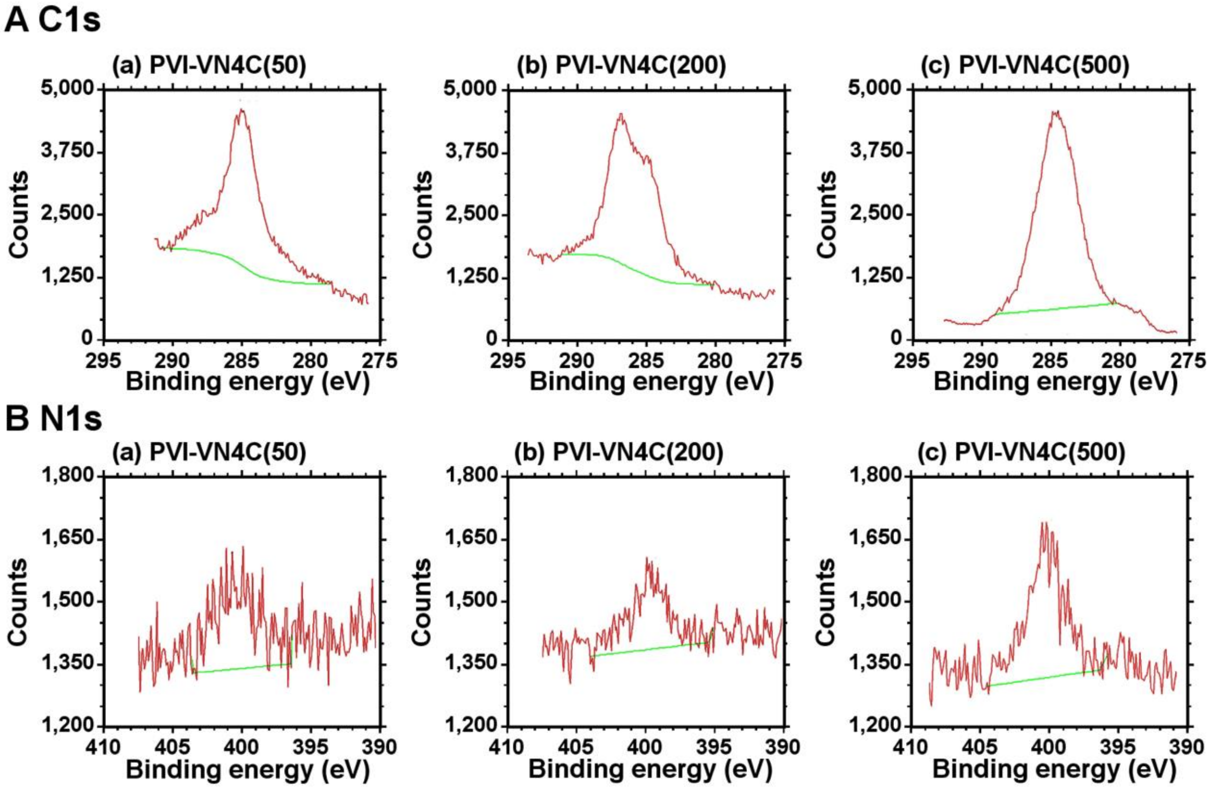

Figure S1. Surface analysis of PVI-VN4C hydrogels. (A) High-resolution XPS spectra of the C 1s peaks on the surfaces of the (a) PVI-VN4C(50) hydrogels, (b) PVI-VN4C(200) hydrogels and (c) PVI-VN4C(500) hydrogels. (B) High-resolution XPS spectra of the N 1s peaks on the surfaces of the (a) PVI-VN4C(50) hydrogels, (b) PVI-VN4C(200) hydrogels and (c) PVI-VN4C(500) hydrogels.
